# Cytogenetic maps of homoeologous chromosomes A _h_01 and D _h_01 and their integration with the genome assembly in *Gossypium hirsutum*

**DOI:** 10.3897/CompCytogen.v11i2.12824

**Published:** 2017-06-19

**Authors:** Yuling Liu, Zhen Liu, Renhai Peng, Yuhong Wang, Zhongli Zhou, Xiaoyan Cai, Xingxing Wang, Zhenmei Zhang, Kunbo Wang, Fang Liu

**Affiliations:** 1 Anyang Institute of Technology, Anyang, Henan, 455000, China; 2 State Key Laboratory of Cotton Biology / Institute of Cotton Research of Chinese Academy of Agricultural Science, Anyang, Henan, 455000, China

**Keywords:** cotton, BAC, FISH, physical map, draft genome assembly

## Abstract

Cytogenetic maps of *Gossypium hirsutum* (Linnaeus, 1753) homoeologous chromosomes A_h_01 and D_h_01 were constructed by fluorescence *in situ* hybridization (FISH), using eleven homoeologous-chromosomes-shared bacterial artificial chromosomes (BACs) clones and one chromosome-specific BAC clone respectively. We compared the cytogenetic maps with the genetic linkage and draft genome assembly maps based on a standardized map unit, relative map position (RMP), which allowed a global view of the relationship of genetic and physical distances along each chromosome, and assembly quality of the draft genome assembly map. By integration of cytogenetic maps with sequence maps of the two chromosomes (A_h_01 and D_h_01), we inferred the locations of two scaffolds and speculated that some homologous sequences belonging to homoeologous chromosomes were removed as repetitiveness during the sequence assembly. The result offers molecular tools for cotton genomics research and also provides valuable information for the improvement of the draft genome assembly.

## Introduction

The genus *Gossypium* (Linnaeus, 1753) includes approximately 47 diploid species (2n = 2x = 26) that are divided into eight genome groups, named as A-G and K genome (Endrizzi et al. 1985, Wendel et al. 2012). Ancient hybridization between A and D diploids resulted in a new allopolyploid (AD) (2n = 4x = 52) lineage approximately 1–2 million years ago (Wendel 1989, Li et al. 2015, Zhang et al. 2015, Liu et al. 2015, Yuan et al. 2015). As the most important natural fiber crop in the world, four *Gossypium* species were independently domesticated for their long, spinnable, epidermal seed trichomes, which include *G. hirsutum* (Linnaeus, 1753) (AD_1_), *G. barbadense* (Linnaeus, 1753) (AD_2_), *G. herbaceum* (Linnaeus, 1753) (A_1_) and *G. arboreum* (Linnaeus, 1753) (A_2_). Among the four species, *G. hirsutum* (AD_1_) provides more than 90% of the world’s cotton fiber production (Wendel and Cronn 2003). Moreover, as a typical polyploid species, cotton is a model system for studying polyploidization. So dissecting the cotton genome is important for facilitating advances in crop germplasm development and utilization, as well as understanding of other polyploid crops. At present, sequencing *Gossypium* species genomes is ongoing in full swing with successively draft maps of whole genome in wild and cultivated cotton species (Paterson et al. 2012, Wang et al. 2012, Li et al. 2014, 2015, Zhang et al. 2015, Liu et al. 2015,Yuan et al. 2015). It is expected that new genome assemblies will soon became available. However, a high level of sequence conservation between homoeologous genomic regions makes it difficult to annotate and assemble whole-genome sequences in allotetraploid species including cotton and wheat (Wang et al. 2010), which may result in many gaps and blurred chromosome scaffolds in the draft genome, and access to high-quality assembly sequence still has a long way to go. Therefore, it is necessary to carry out the relevant basic research work on cotton genome research to help for genome sequence assembly.

The uneven distribution of recombination events on chromosomes results in divergence between genetic distance and physical distance, which limits the application of genetic map in guiding genome sequence assembly and map-based cloning (Sun et al. 2013). A cytogenetic map, which can integrate genetic loci into physical location of chromosome, has great potential to help in the assembly of genome sequence. Fluorescence *in situ* hybridization (FISH), which allows direct mapping of DNA sequence on chromosome, has been widely used in the study of different plants as an important tool for constructing cytogenetic maps (Jiang and Gill 2006). At present, physical maps based on high resolution FISH in many crops have been reported, such as maize (Figueroa and Bass 2012), rice (Cheng et al. 2001, Kao et al. 2006), *Brassica* (Linnaeus, 1753) (Xiong et al. 2010), tomato (Koo et al. 2008, Szinay et al. 2008), potato (Tang et al. 2008), bean (Fonsêca et al. 2010), cucumber (Han et al. 2011, Sun et al. 2013).

Tetraploid cotton contains too many chromosomes (2n = 4x = 52) and it is difficult to prepare chromosomes due to large amounts of secondary metabolites in cells. So research on cotton cytogenetic maps has lagged behind other crops. Moreover, previous cotton FISH mapping was mainly limited to the use of repetitive DNA (Hanson et al. 1996, Ji et al. 2007), the chromosome-specific bacterial artificial chromosomes (BACs) (Wang et al. 2007). To date, there have been only a few cotton cytogenetic maps (Wang et al. 2010, Cui et al. 2015).

Structure analysis of homoeologous chromosomes in allotetraploid cotton plays an important guiding role in sequence assembly, map-based cloning, and so on. Xu et al. (2008) selected homoeologous chromosomes Chr.12 and Chr.26 (12A and 12D) in allotetraploid cotton, which contain important genes related to fiber fuzz, gland development, and male sterility, and constructed their physical maps using the BAC contigs, which provided an important platform for the clone mapping of the important genes. Wang et al. (2010) constructed cytogenetic maps of homoeologous chromosomes 12A and 12D using BAC-FISH, which had guided the next genome sequence assembly to a certain extent (Zhang et al. 2015). Chr.01 and Chr.15 (i.e. A_h_01 and D_h_01) in upland cotton linkages have been shown to be homoeologous chromosomes based on genetic markers, which contain many genes or QTLs related to stress tolerance, fiber development, fiber yield and quality (Said et al. 2013). In this study, the cytogenetic maps of homoeologous chromosomes A_h_01 and D_h_01 of *G. hirsutum* were constructed by FISH using marker-anchored BACs. By using similar relative map position (RMP) units, which was the percentage distance of a locus from the end of the short arm along a given chromosome, we made a comparative analysis between the cytogenetic, the genetic linkage, and draft genome assembly maps of *G. hirsutum* homoeologous chromosomes A_h_01 and D_h_01 preliminarily.

## Material and methods

### Plant materials and BAC library

*G. hirsutum* (Linnaeus, 1753) accession TM-1 was used for cytological studies. BACs used for FISH mapping were identified by screening two genomic BAC libraries derived from *G. herbaceum* (Linnaeus, 1753) *var. africenum* (Gao et al. 2013) and *G. barbadense* (Linnaeus, 1753) Pima 90-53 (kindly provided by Prof. Zhiying Ma of Hebei Agricultural University). The chromosome-specific BAC clones for *G. hirsutum* A_h_01/D_h_01 were kindly provided by Prof. Tianzhen Zhang of Nanjing Agricultural University, The simple sequence repeat (SSR) markers used for BAC screening were selected from a whole genome marker map (WGMM) (Wang et al. 2013) and a genetic map (Yu et al. 2011).

### BAC library screening

The screening was performed using bacteria liquid-PCR according to the protocol previously described (Cheng et al. 2012).

### Chromosome preparation and FISH

Chromosome preparation and FISH were conducted according to the previous protocols (Gan et al. 2011). In order to reduce the interference from the background signals, heat-shock-interrupted (1.5 mL Eppendorf tube filled with 100 μl genome DNA was placed in sterilization pot with 105°C for 8 min) cotton genome DNA fragments with size from 200 bp to 800 bp were used as blocking DNA. BAC-DNA used to label probes was isolated using Plasmid Miniprep Kit (Biomiga) according to the handbook. Biotin- and digoxigenin-labeled probes were detected using rhodamine-conjugated anti-digoxigenin and fluorescein-conjugated avidin (Roche Diagnostics, USA), respectively. Chromosomes were counter-stained with 4, 6-diamidino-2-phenylindole (DAPI, Sigma, USA) and antifade (Vector, USA) under a cover-slip.

### Image analysis

Slides were examined under a Zeiss Imager M1 microscope. Images were captured and merged using MetaSystems isis software with a CCD camera (MetaSystems CoolCube 1) attached to a Zeiss Imager M1 microscope. To determine physical positions of signals, only chromosomes without apparent morphological distortion were introduced and their physical positions of signals were measured using MetaSystems isis. Final image adjustments were performed using Adobe Photoshop CS3 software.

### Comparative mapping using standardized map units

The RMP unit was used as standardized map unit for comparative analysis between different types of maps. The RMP values for the SSR linkage map were the percentage from the genetic location (cM) of each locus along the total length (cM) of the corresponding linkage group. The RMP values of the cytogenetic map were the percentage of the distance (μm) from the FISH signal site to the end of the short arm showed relative to the total length of the chromosome (μm) (Sun et al. 2013). In order to determine the genomic locations (bp) of each BAC clones, the primer sequences of BACs-corresponding SSR markers were obtained from the database Cotton Marker Database (http://www.cottonmarker.org/), then according to Electronic PCR command line tools (Version 2.3.12), e-PCR was run against the *G. hirsutum* (AD_1_) genome NAU-NBI Assembly (https://www.cottongen.org/organism/*Gossypium*/*hirsutum*) according to the default parameters. The RMP values for the *G. hirsutum* draft genome assembly map were calculated from the genomic location (bp) of each locus along the physical length of chromosomes A_h_01 and D_h_01. These RMP values were used to produce the comparative map alignments.

## Results

### Screening of SSR markers

To construct the cytogenetic maps of chromosomes A_h_01 and D_h_01 of *G. hirsutum*, an initial set of 47 SSR markers shared by both chromosomes of A_h_01 and D_h_01 from a whole genome marker map (WGMM) (Wang et al. 2013, Rong et al. 2004) and a genetic map (Yu et al. 2011) were used to screen two BAC libraries of *G. herbaceum var. africenum* and *G. barbadense* Pima 90-53. Based on the WGMM, the SSR markers were distributed along the linkage group of chr.15 (D_h_01) from 0.6 cM (CIR009) to 176.3 cM (CIR110) (Table 1). In total, 84 positive BAC clones were identified based on the result of BAC libraries screening (Table 2). Due to abundance of repetitive sequence in cotton genome, by dual-color FISH with the chromosome-specific BAC clones 52D06 (A1) and 48F11 (D1) as controls, only 12 BAC clones were selected for FISH mapping which produced little or no background signal when hybridized to *G. hirsutum* chromosomes with the aid of blocking DNA.

**Table 1.** Information of selected SSR markers based on the WGMM*^1.^

SSR	BAC	Loc. in D-genome sequence			Chr.15 cM	RMP (%)*^2^	Loc. in tetraploid	
		Chr.	Start bp	End bp				
NAU2015	305A19	Chr02	61962135	61962910	12.6	7.14	Chr.01	Chr.15
NAU3254	348I20	Chr02	60694684	60699332	29.1	16.49	Chr.01	Chr.15
NAU2474	144E04	Chr02	59155451	59156001	39.5	22.39	Chr.01	Chr.15
NAU3433	64M24	Chr02	55462914	55463585	53.6	30.38	Chr.01	Chr.15
BNL2921	400N03	Chr02	27353761	27353982	73.3	41.55	Chr.01	Chr.15
NAU4891	118G12	Chr02	15429614	15428837	86.2	48.86	Chr.01	Chr.15
NAU3135	85P13	Chr02	11717323	11717890	90.2	51.13	Chr.01	Chr.15
BNL3888b	164I21	Chr02	11188812	11189229	90.3	51.19	Chr.01	Chr.15
BNL3580	421E24	Chr02	7879846	7880283	93.4	52.94	Chr.01	Chr.15
NAU4044	400L15	Chr02	2312144	2313542	111.5	63.20	Chr.01	Chr.15
HAU076	378J07	--*3	--	--	--	--	Chr.01	--
TMB0062	423C18	--*^3^	--	--	--	--	Chr.01	--

Note: *^1^, WGMM, whole-genome marker map. *^2^, RMP, relative map position, it refers to the percentage of marker’s cM value accounting for chromosome’s total cM value. *^3^, SSR derived from a tetraploid genetic map (Yu et al. 2011).

**Table 2.** BAC clones screened from two BAC libraries.

SSR markers	BAC library	Screened BAC clones
HAU2861	1*	22K17; 22L15; 22L18; 67J23; 75D24; 75E24; 108E08; 108E24; 130M09; 151C24; 151E18
NAU3433	1*	41J08; 41K08; 46K02; 64M20; 64M24; 78G20; 78H20
NAU3053	1*	22K18; 22L17; 67I12; 75C23; 75E24;107P10; 107P24
NAU4891	1*	50H19; 51C14; 51H12; 56J17; 118G11; 118G12
Gh649	1*	99L01; 136O19; 136P17
NAU2095	1*	52B01
Gh216	1*	50P23; 57I23; 79A06; 79A12; 79B07; 101K10; 101K12; 146P05
NAU5163	2*	141H01; 158M07; 158N09; 158L08; 159L07; 159L08
BNL3888b	2*	164I21; 164I22
NAU3254	2*	348I18; 348I20; 348I21; 348H17; 348J19
CIR049	2*	256N07
BNL2921	2*	400N03; 400L02
BNL3580	2*	421E24
NAU2015	2*	305A19
NAU4044	2*	400L15
NAU2474	2*	144E04; 165B11
NAU3135	2*	85P13; 377G04; 377H05; 247P16; 247P17; 325M09; 325M10
TMB0062	2*	298N21; 403A13; 423C18; 423C19; 424A12
HAU076	2*	249G03; 249G04; 249I5; 325N10; 378J07; 398J05; 398H05; 249G05

Note: 1* BAC library *G. herbaceum var. africenum* 2* BAC library *G. barbadense* Pima 90-53

### FISH identification

**Figure 1.**
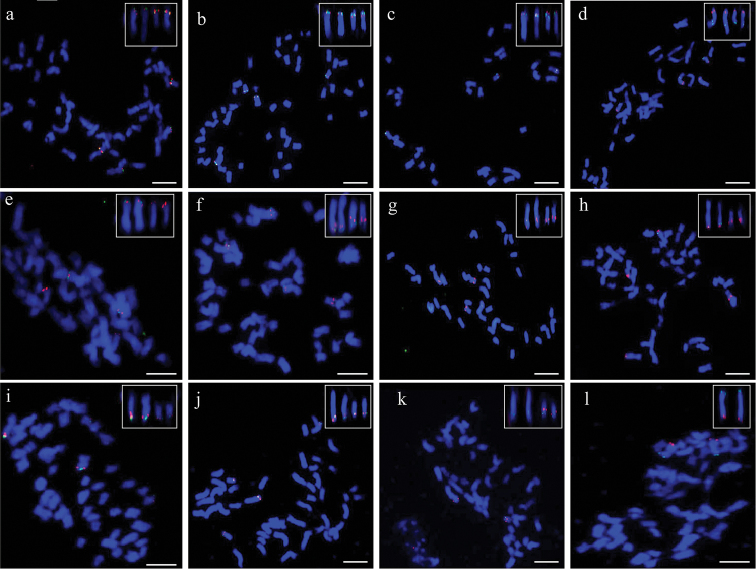
The order of two BACs on metaphase chromosome of *G. hirsutum* (AD_1_) TM-1 using Dual-color-FISH. **a** 305A19(green)/348I20(red) **b** 305A19 (green)/64M24(red) **c** 144E04 (red)/64M24(green) **d** 64M24 (red)/400N03(green) **e** 305A19 (red)/378J07(green) **f** 118G12(red)/164I24(green) **g** 423C18 (red)/400L15(green) **h** 85P13 (green)/400L15(red) **i** 85P13 (red)/421E24(green) **j** 85P13 (red)/164I24(green) **k** D1 (red)/118G12(green) **l** 378J07 (green)/ A1 (red). Bar = 5 µm.

By dual-color FISH on mitotic chromosomes, the order of the two BACs was determined along the chromosomes based on the genetic positions of their corresponding SSR markers. Results showed, among the 12 positive BAC clones, 11 BAC clones were homoeologous-specific BACs because they generated signals on both chromosomes of A_h_01 and D_h_01, indicating sequence homology between these BACs retained in A_h_01 and D_h_01 (Fig. 1a-k). One BAC clone 378J07, derived from SSR HAU076, only had one pair of FISH signals on chromosome A_h_01, which had collinearity with the chromosome A_h_01-specific BAC clone A1 (52D06) (Fig. 1l). Based on these results, the relative position of all probes can be preliminarily distinguished along the mitotic metaphase chromosomes.

### Construction of the cytogenetic maps

**Figure 2.**
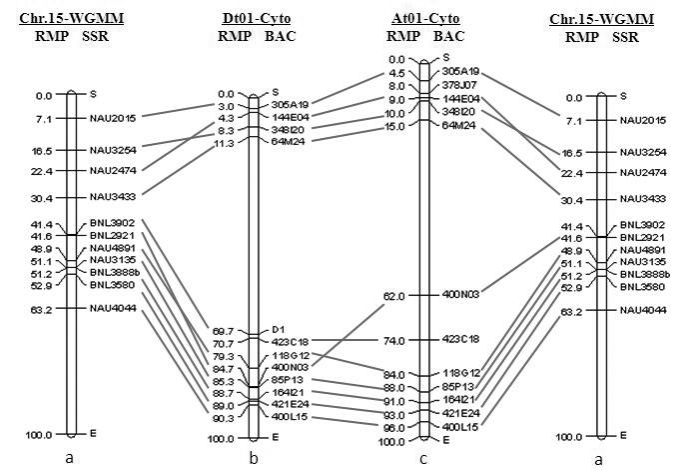
Comparison of positions of BACs in cytogenetic maps of *G. hirsutum* A_h_01/D_h_01 with genetic positions of SSR markers **a** Positions of SSR markers based on WGMM; **c, b** Cytogenetic maps of *G. hirsutum* A_h_01/D_h_01.

The genetic distances of SSR markers associated with the corresponding BACs were also converted into the relative positions in the corresponding linkage map (Fig. 2a). In order to confirm the physical position of each clone, FISH signal of each BAC clone was measured in 5-8 cells with clear chromosome spreads and the RMP of FISH signals were computed (Table 3). Based on the data, the cytogenetic maps of the homoeologous chromosomes D_h_01 and A_h_01 were constructed (Fig. 2b, c). The order of individual BACs along the chromosome was generally collinear with the order of the corresponding SSR markers along the linkage map, except for a few closely linked loci, 144E04 (NAU2474) and 348I20 (NAU3253), 118G12 (NAU4891) and 400N03 (BNL2921), which displayed changes in the order between the genetic markers and BAC locations (Fig. 2a, b). Moreover, the BACs showed better concordance in the orders and positions between the two cytogenetic maps of the homoeologous chromosomes A_h_01 and D_h_01, except for 400N03 (BNL2921) (Fig. 2b, c), which suggests a rearrangement between the A_h_01 and D_h_01 homoeologous chromosomes in the process of evolution. A significant difference between the two types of maps was viewed, that is, the markers flanking the middle region were separated by short genetic distance but long physical distance. For example, the genetic distance between markers NAU3433 and BNL2921 is 11.2% of total genetic distance of chromosome 15 (D_h_01), but the physical distances between these two markers is 59.4% of the total length of the chromosome D_h_01 (Fig. 2a, b).

**Table 3.** Physical locations of FISH-mapped BACs in *G. hirsutum* draft genome assembly and cytogenetic map.

BAC	SSR marker	Loc. in AD_1_^*1^ draft genome				Loc. in AD_1_ Cytogenetic map ^*3^	
		No. of chromosome	Start (bp)	End (bp)	RMP(%)^*2^	D_h_01 RMP(%)	A_h_01 RMP(%)
305A19	NAU2015	D_h_01	60681011	60681490	1.26	3.00±0	4.51±0.41
378J07	HAU076	A_h_01	96488204	96488397	3.40	/	8.01±0.48
144E04	NAU2474	D_h_01	57722851	57723034	6.07	4.33±0.47	/
		scaffold183_A01	19925	20108	/	/	9.01±1.25
348I20	NAU3254	D_h_01	59322542	59322834	3.47	8.33±0.47	10.02±0.51
64M24	NAU3433	A_h_01	90268406	90268610	9.63	/	15.00±0.47
		D_h_01	53813626	53813830	12.44	11.33±1.24	/
400N03	BNL2921	A_h_01	40133025	40133182	59.82	84.66±0.47	61.99±0.94
423C18	TMB0062	A_h_01	17562250	17562499	82.42	70.66±5.24	74.11±0.36
118G12	NAU4891	A_h_01	17991434	17991731	81.99	79.33±4.49	84.01±1.10
85P13	NAU3135	A_h_01	11722545	11722728	88.26	/	88.07±0.19
		D_h_01	9387192	9387374	84.73	85.33±0.47	/
D1	BNL3902^*4^	D_h_01	26803236	26803427	56.38	69.66±0.94	/
164I21	BNL3888b	A_h_01	11084705	11084886	88.90	88.66±1.69	90.98±0.27
421E24	BNL3580	A_h_01	7078093	7078309	92.91	89.00±1.41	92.99±0.65
400L15	NAU4044	A_h_01	2245730	2245951	97.75	/	96.01±1.19
		scaffold3710_D01	109956	110177	/	90.33±1.24	/

Note: *1, the AD_1_-NBI draft genome (Zhang et al. 2015); *2, RMP: relative map position, in cytogenetic map, it refers to the percentage of the distance (μm) from the FISH signal site to the end of the one arm accounting for the total length of the chromosome; in sequence map, it refers to the percentage of the sequence location of the corresponding SSRs of BACs accounting for the total length of the chromosome (A_h_01 = 99884700 bp, D_h_01 = 61456009 bp); *3, 5-8 cells were used for measurement; *4 corresponding SSR of D_h_01-specific BAC.

### Integration and analysis of BACs positions across the cytogenetic and genome assembly maps

**Figure 3.**
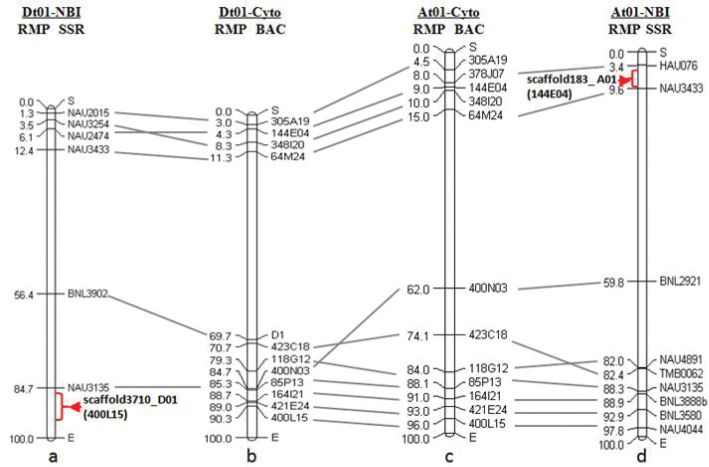
Integrated cytogenetic /genome assembly maps of *G. hirsutum* A_h_01/D_h_01. **a** Relative map position of BACs mapping to D_h_01 of the AD_1_-NBI draft genome **b** Cytogenetic map of *G. hirsutum* Chromosome D_h_01 based on 12 BAC clones **c** Cytogenetic map of *G. hirsutum* A_h_01 based on 12 BAC clones **d** Relative map position of BACs mapping to A_h_01 of the AD_1_-NBI draft genome. Arrow-head in a and d represent the locations of scaffold3710_D01 and scaffold183_A01 in the draft genome (AD_1_-NBI) respectively.

To compare our cytogenetic maps directly to the draft genome assembly map (Zhang et al. 2015), the corresponding SSR primers of the BAC clones were mapped to the draft genome sequence by e-PCR, and the relative positions of the SSRs were calculated according to the e-PCR results (Table 3). Based on the above data, we integrated the cytogenetic maps with the genome sequence maps of the homoeologous chromosomes A_h_01 and D_h_01 to compare their distributions (Fig. 3). The alignments allowed a global view of the relations between the chromosomal positions and physical positions in draft genome map of the BAC clones. The number of BACs mapped on each pseudo-chromosome in the draft genome assembly map was significantly less than that on the corresponding cytogenetic maps (six to twelve on D_h_01, nine to twelve on A_h_01) (Fig. 3). Of the eleven homoeologous-chromosomes-shared BACs based on cytogenetic maps, four BACs’ corresponding SSR markers (NAU3433, NAU3135, NAU2474 and NAU4044) were simultaneously mapped on the two corresponding chromosomes in *G. hirsutum* draft genome assembly. The others were only mapped on one of the chromosome A_h_01 or D_h_01 respectively. NAU2474 was mapped on the chromosome D_h_01 and scaffold183_A01 of the draft genome assembly by e-PCR. Its corresponding BAC clone 144E04 was FISH mapped on chromosome A_h_01 (RMP 9.01%) and D_h_01 (RMP 4.33%) in cytogenetic maps. NAU4044 was mapped on the chromosome A_h_01 and scaffold3710_D01 of the draft genome assembly by e-PCR. Its corresponding BAC clone 400L15 was FISH mapped on chromosome A_h_01 (RMP 96.01%) and D_h_01 (RMP 90.33%) in cytogenetic maps. Based on these comparison results, the locations of the two scaffolds in the draft genome assembly were determined approximately. That is, scaffold183_A01 (size 55529 bp) located between the SSR markers HAU076 and NAU3433 on the chromosome A_h_01, i.e., the relative position between 3.4% and 9.6% (sequence loci from 90268610 bp to 96488204 bp) (shown by arrow Fig. 3d). Scaffold3710_D01 (size 191022 bp) locates near the end of the chromosome D_h_01, i.e., the outer of the relative position 84.7% (sequence loci from 6145600 bp to 9387374 bp) (shown by arrow Fig. 3a).

## Discussion

### Integration of the genetic and cytogenetic maps of homoeologous chromosomes A_h_01 and D_h_01

In cotton, more than 30 genetic maps have been published, including several integrated maps with higher marker density (Yu et al. 2010, Yu et al. 2011, Blenda et al. 2012), and a whole-genome marker map (WGMM) by integrating publicly available sequence tagged DNA markers with the cotton D-genome sequence (Wang et al. 2013). Undeniably, they are a foundational tool and resources for marker-assisted selection and genomic studies. But the linkage maps provide little information about physical locations, distributions, distances, and sometimes orientations of genetic markers. Cytogenetic maps encompassing the information from both genetic maps and cytological maps, can relate the markers mapped across linkage groups to cytological position on chromosomes. Using a set of marker-anchored BACs, we developed the cytogenetic maps of homoeologous chromosomes Ah01 and Dh01 in *G. hirsutum*. The comparative map alignments revealed a significant disproportion between genetic and physical distances in the pericentromeric region, such as, the distance between markers NAU3433 and BNL2921 with 11.2 RMP(Fig. 2a) but on the cytogenetic map with 59.4 RMP (Fig. 2b). The reduction of recombination around the chromosome centromere is a common feature and the region of recombination suppression correlates directly with sizes of centromeric heterochromatic regions (Sun et al. 2013). So this implies larger region of suppressed recombination was detected in the pericentromeric region of chromosome D_h_01. Moreover, the orders of most genetic markers are collinear with corresponding BAC locations although several closely linked loci in D_h_01 display inconsistent orders or locations compared with those in BAC FISH maps.

In total, the integrated genetic and cytogenetic maps can serve as a template to facilitate sequence assembly, because the maps provided information on the distribution of genetic markers across chromosomes and the linkage gaps derived from recombination suppression.

### Homologous relationships between chromosomes A_h_01 and D_h_01

As a typical allotetraploid, which contains two sub-genomes originating from related ancestor species with different genome sizes, *G. hirsutum* has been studied on its homoeologous chromosomes for a long time. Results revealed that fragment additivity (Liu et al. 2001), the independence of evolution of duplicated genes (Cronn et al. 1999), conservation in gene content, order, and spacing (Grover et al. 2004, 2007) between the homoeologous chromosomes, as well as the potential mechanisms for genome-size variation in the homoeologous chromosomes (Wang et al. 2010). Here, we constructed the cytogenetic maps of homoeologous chromosomes A_h_01 and D_h_01 using shared-markers-anchored BACs. By comparison analysis of BACs’ positions, consistent orders of FISH signals were viewed in both homoeologous chromosomes, except for one BAC clone 400N03, which showed obvious location discrepancy in the homoeologous chromosomes (RMP 62% in A_h_01 and 84.7% in D_h_01). The discrepancy may be caused by a chromosomal rearrangement in this region during a certain period of polyploidization. In addition, better collinearity of ten of eleven shared BACs between the homoeologous chromosomes suggests that there remains a generally high level of sequence conservation between homoeologous chromosomes A_h_01 and D_h_01, though polyploidization occurred about 2 MYA (Cronn et al. 2002, Seelanan et al. 1997, Wendel 1989).

### Integration of the cytogenetic maps and the cytogenetic and genome assembly maps

The e-PCR can be used to search for sub-sequences that closely match the primers of SSRs, which can help to identify the genome positions of SSRs within the reference genome sequence (McCouch et al. 2002, Li et al. 2015). In this study, we identified the genome positions of thirteen SSRs using e-PCR. Results showed the length and position of the target sequence for each pair of primers against the reference genome sequence were consistent with the initial selection, which ensured the accuracy of the next relative position calculation and comparative analysis.

Mis-assemblies are common when draft genome sequences have been generated by de novo assembly of sequences obtained with NGS technologies (Meader et al. 2010, Alkan et al. 2011). Since the assembly of *G. hirsutum* was done using the SOAPdenovo software, the final assembly comprised 265,279 contigs and 40,407 scaffolds (Zhang et al. 2015), so mis-assembled scaffolds may exist in the draft genome. On the other hand, there are a generally high level of sequence conservation between homoeologous genomic regions in allotetraploid species including cotton and wheat (Zhao et al. 2012, Brenchley et al. 2012), it is difficult to annotate and assemble whole-genome sequences. Since the cytogenetic map can reflect the true position of the DNA sequence in the chromosome, so it has some significance for verification and correction of the genome assembly. In the process of genome sequencing and sequence assembly, the cytogenetic map plays a role in filling the sequencing gaps, correcting assembly errors, evaluating the quality of assembly, achieving more scaffolds and contigs chromosomal localization and orientation. Wang et al. mapped 32 BAC clones to some of the homologous chromosomes 12A and 12D of upland cotton by FISH, and constructed the high resolution cytogenetic map of the two chromosomes (Wang et al. 2010). Through the integration of genetic loci and physical sites, considerable variations in the composition, structure and size of the two homoeologous chromosomes were viewed, which play an important role in the sequencing and sequence assembly of *G. hirsutum* (Wang et al. 2010; Zhang et al. 2015). By comparison of the distributions of fosmid clones on the cucumber draft genome assembly map and cytogenetic map, the accuracy and coverage of the draft genome assembly map were evaluated (Sun et al. 2013).

Here, we constructed the cytogenetic maps of homoeologous chromosomes A_h_01 and D_h_01 using shared-BACs. By integration of cytogenetic maps and the cytogenetic and genome assembly maps, we identified the positions of two scaffolds in chromosome (Fig. 3a, d). Among the eleven shared-BACs in the cytogenetic maps of chromosomes A_h_01 and D_h_01, only four (accounting for 36.36%) had hits both in two corresponding pseudo-chromosome in the draft genome assembly map, the others were only mapped on one of the chromosome A_h_01 or D_h_01 respectively. It may be that some homologous sequences were removed as repeats, and only partial sequences information with homology were assembled on one of the two homoeologous chromosomes during the assembly process.

## Conclusions

We demonstrated concordant orders and RMP of markers between the sequence map and physical map based on FISH. By integration of cytogenetic maps with sequence maps of the two chromosomes, we inferred the locations of the two scaffolds, and speculated some homologous sequences belonging to homoeologous chromosomes were removed as repetitiveness during the process of sequence assembly. Our study not only offers molecular tools for cotton genomics research, but also provides valuable information for the improvement of the draft genome assembly.
